# Atomic-scale observation of premelting at 2D lattice defects inside oxide crystals

**DOI:** 10.1038/s41467-023-37977-w

**Published:** 2023-04-20

**Authors:** Hye-Sung Kim, Ji-Sang An, Hyung Bin Bae, Sung-Yoon Chung

**Affiliations:** 1 Department of Materials Science and Engineering, Korea Advanced Institute of Science and Technology, Daejeon, 34141 Korea; 2 KAIST Analysis Center, Korea Advanced Institute of Science and Technology, Daejeon, 34141 Korea; 3 Present Address: Korea Institute of Energy Research, Daejeon, 34129 Korea

**Keywords:** Structure of solids and liquids, Phase transitions and critical phenomena, Characterization and analytical techniques

## Abstract

Since two major criteria for melting were proposed by Lindemann and Born in the early 1900s, many simulations and observations have been carried out to elucidate the premelting phenomena largely at the crystal surfaces and grain boundaries below the bulk melting point. Although dislocations and clusters of vacancies and interstitials were predicted as possible origins to trigger the melting, experimental direct observations demonstrating the correlation of premelting with lattice defects inside a crystal remain elusive. Using atomic-column-resolved imaging with scanning transmission electron microscopy in polycrystalline BaCeO_3_, here we clarify the initiation of melting at two-dimensional faults inside the crystals below the melting temperature. In particular, melting in a layer-by-layer manner rather than random nucleation at the early stage was identified as a notable finding. Emphasizing the value of direct atomistic observation, our study suggests that lattice defects inside crystals should not be overlooked as preferential nucleation sites for phase transformation including melting.

## Abstract (web summary)

No experimental observations have been reported to clarify how a melting transition proceeds inside a crystal. Here the authors demonstrate that melting is initiated at two-dimensional faults inside BaCeO_3_ crystals below the melting temperature in a layer-by-layer manner.

## Introduction

A crystalline solid melts into a liquid phase, showing typical first-order phase transformation behavior of significant discontinuities in the thermodynamic variables, such as molar volume and entropy, at the melting point. As in other phase transformations via a heterogeneous nucleation process, it has been accepted that melting is preferentially initiated at the crystal surfaces^1–5^, grain boundaries^6–8^, and dislocations^9,10^. In particular, as reviewed in ice^11,12^, this local loss of crystallinity followed by the subsequent formation of a liquid layer below the bulk melting temperature is called premelting of a crystal. While numerous studies have been reported to elucidate the notable features during crystallization of various materials, few attempts at experimental observation on melting at atomic-column resolution in inorganic crystals have been made since the early empirical work by Lindemann in 1910 based on the atomic mean square displacement as a criterion of melting^13^.

Direct imaging and dynamic simulations of melting have several limitations. First, most observations of melting have been confined to the interfaces encompassing surfaces and grain boundaries^1,3–6^. As a result, experimental evidence of whether lattice defects inside a crystal indeed act as preferential sites for premelting remains elusive, even though Frenkel theoretically suggested that the formation of vacancies and interstitials is required for melting in a crystal^14^. Moreover, unless the defect sites in the crystal lattice are clearly visualized, it is considerably challenging to trace where the melting begins and how it evolves further. In addition, computer-aided simulations and experimental investigations on crystal melting have been largely performed in single-component materials to avoid complexity stemming from multiple compositions and sophisticated crystal structures^15–19^. Consequently, no atomistic details on the premelting have been reported in complex ionic crystals.

**Fig. 1:**
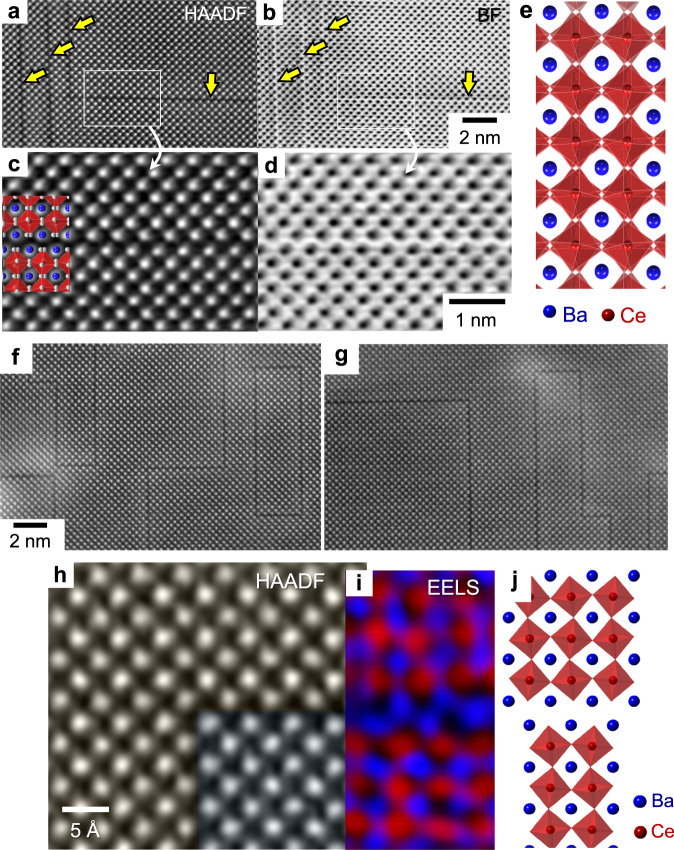
Structure and composition at RP faults in BaCeO_3_. **a–d** A pair of HAADF and BF STEM images **a**, **b** is provided along with the enlargements **c**, **d**, indicating the image contrast for the presence of faults, as denoted by yellow arrows. The overall crystal structure of BaCeO_3_ is shown in the right-hand column. **e** The crystal structure of BaCeO_3_ is presented. **f**, **g** These two lower-magnification images demonstrate the various distribution of faults in a grain. **h** The image contrast of the fault is not clearly distinguished in the margined image. **i** However, the atomic-column-resolved EELS mapping directly demonstrates the two consecutive Ba−Ba columns at the fault plane. **j** An atomic array illustration along with the map is also provided for verification.

Dealing with perovskite-type BaCeO_3_ as a model oxide in this work, we provide the atomic-scale experimental evidence of premelting at lattice defects inside oxide crystals. This observation thus demonstrates that the lattice defects inside a crystal are significant origins of the vibrational and elastic instability triggering melting, as suggested by both Lindemann and Born criteria^13,20^. To scrutinize the premelting aspects at an atomic level, the selection of defect sites is of great significance for successful and straightforward observation. We thus exploit two-dimensional (2D) homologous faults, which are not interfaces (such as surfaces and grain boundaries) but rather are internal lattice defects consisting of two consecutive [BaO] layers along the {001} planes inside the crystal, as will be described in more detail in Fig. 1. One of the key features that should not be overlooked in the present study is that premelting takes place in a layer-by-layer manner, directly demonstrating melting of the [BaO] layers followed by the neighboring [CeO_2_] layers. We also identify that melting further proceeds along with the atomically rough interface between the crystal and the liquid layer as the annealing temperature is elevated. Experimentally verifying the crucial role of lattice defects in premelting inside a crystal at atomic resolution, our work highlights the value of direct probing to unveil the phase transformation behavior in sophisticated condensed-matter systems.

## Results

### Observation of Ruddlesden−Popper faults

As recently visualized in many ABX_3_-type materials^21^, 2D homologous Ruddlesden−Popper (RP) faults of [AX]−[AX] interleaved layers along the [100] direction in a cubic framework are frequently observed in a variety of perovskite oxides and halides^22–27^. Figure 1a, b present a pair of high-angle annular dark-field (HAADF) and bright-field (BF) scanning transmission electron microscopy (STEM) images showing the presence of RP faults in a BaCeO_3_ crystal from a polycrystalline sample sintered at 1330 °C. A pair of enlarged images for the location denoted by a white rectangle is also provided in Fig. 1c, d along with the crystal structure illustration in Fig. 1e. STEM images at a lower magnification are also shown in Fig. 1f, g for the fault distribution (see Supplementary Fig. 1 for an additional set of STEM images of the fault distribution). The atomic number (*Z*) contrast between Ba (*Z*_Ba_ = 56) and Ce (*Z*_Ce_ = 58) in the HAADF mode is considerably low. Therefore, it is somewhat difficult to readily recognize the presence of two consecutive [BaO] layers in the magnified HAADF images at a first glance in Fig. 1h, although a sufficient contrast indicative of the fault planes is identified in the pair of low-magnification images in Fig. 1a, b, as denoted by yellow arrows. However, atomic-column-resolved chemical mapping by electron energy-loos spectroscopy (EELS)^21,28^ in STEM straightforwardly confirms the formation of RP faults. As shown in the EELS map in Fig. 1i, the consecutive Ba columns (blue) across the fault plane (Fig. 1j) are directly verified. A chemical map obtained by energy dispersive x-ray spectroscopy (EDS) at an atomic scale^29–32^ is also provided in Supplementary Fig. 2 for confirmation.

**Fig. 2:**
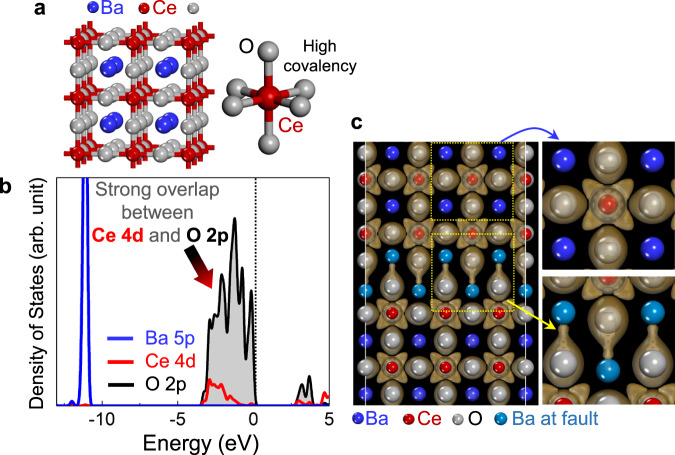
Bond characteristics at RP faults in BaCeO_3_. **a** The crystal structure of BaCeO_3_ is presented. **b** This DOS plot indicates a strong overlap between the O 2*p* and Ce 4*d* states in contrast to no overlap of the Ba 5*p* states with the electronic states of oxygen below the Fermi level. Consequently, a high degree of covalency in Ce−O bonding is represented. **c** In good agreement with the DOS plot, A high electronic density between Ce and O is visualized in this isosurface contour map, as specifically illustrated in the Ce−O contour in the upper right panel. In contrast, no substantial electron density is noted between Ba and O at the fault plane in the lower right panel.

Prior to specific STEM observations, it is necessary to understand the crystal structure and the different nature of chemical bonding between Ba−O and Ce−O in BaCeO_3_. As illustrated in Fig. 2a, the basic framework of ABO_3_ perovskites can be considered a face-centered-cubic (FCC) derivative structure in which the large A cation ion and oxygen together form an FCC lattice. The smaller B cation occupies the octahedral interstitial sites, having six oxygen anions as the nearest neighbors, while the large A cation locates in the dodecahedral sites. Consequently, the bond length of Ba−O is larger than that of Ce−O. As can be seen in the density of states (DOS) of BaCeO_3_ in Fig. 2b, a strong overlap between the O 2*p* (black curve) and Ce 4*d* (red curves) states in the {CeO_6_} octahedra is identified below the Fermi level (*E*_F_ = 0 eV) in the valence band, whereas the Ba 5*p* states are located far below the O 2*p* states with no overlapping. This indicates a substantial degree of covalent bonding in Ce−O in contrast to Ba−O. Indeed, the significant covalency between Ce and O can be visualized when the distribution of the electron density at each atom is acquired by density functional theory (DFT) calculations. Figure 2c presents the isosurface contour of the electron-density difference in a BaCeO_3_ supercell containing an RP fault. As clarified in the magnified contour maps, there is not a notable distribution of electron density between Ba (sky blue) and O (gray) at the fault plane (lower map), while a high density of electrons between Ce and O can be found (upper map). Consequently, unless Ba atoms are surrounded by stable [CeO_6_] octahedra, the Ba−O bonds at the RP fault plane may be easily breakable, and thereby premelting at these defect sites is anticipated to occur.

### Premelting at the faults inside crystals

**Fig. 3:**
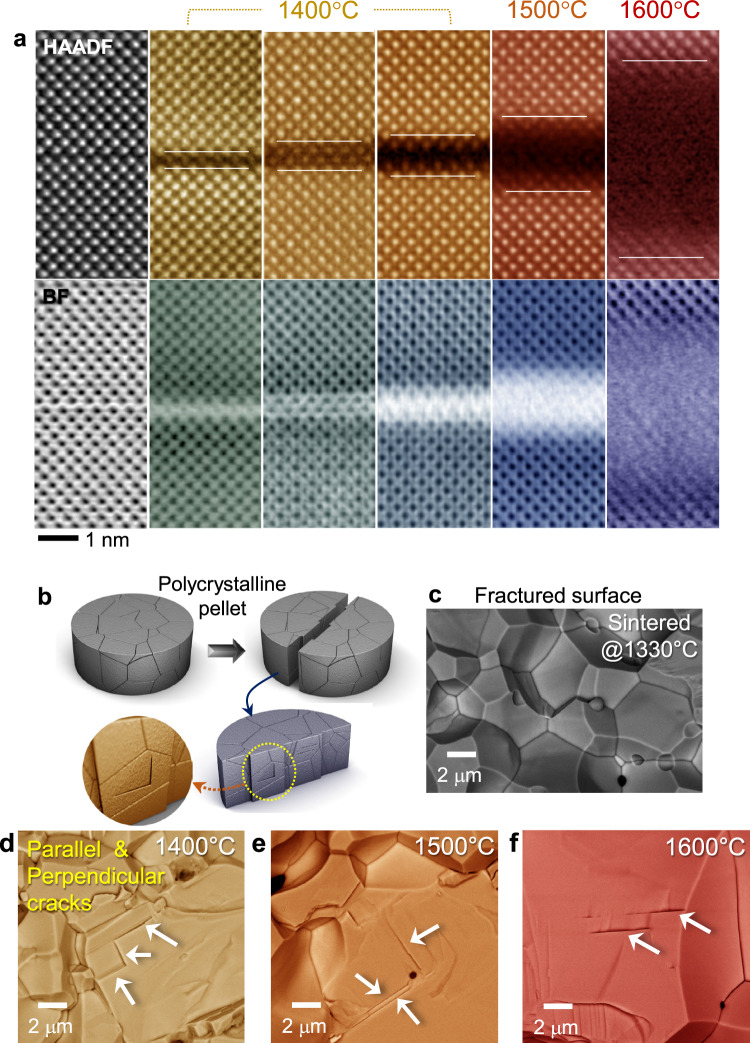
Observation of premelting at the faults. **a** The loss of column contrast by melting is easily observable in both HAADF and BF imaging modes. As denoted by a pair of white lines in each HAADF image, the amorphous layer becomes thicker with increasing the post-annealing temperature. **b** A schematic illustration depicts the observation of the fractured surface by SEM. **c** Before premelting at the faults in the pellet sintered at 1330 °C, the overall microstructure of the fractured surface exhibits typical polycrystalline microstructure consisting of grains and grain boundaries. **d−f** In contrast, micron-level cracks (white arrows) are frequently observed in all the samples post-annealed at 1400 °C (**d**), 1500 °C (**e**), and 1600 °C (**f**), showing the either geometrically perpendicular or parallel configuration.

The sintering temperature to fabricate dense and large-grain polycrystalline BaCeO_3_ for application as proton conducting electrolytes^33–37^ usually ranges from 1400 to 1650 °C below the melting point, ~1740 °C^38,39^. We thus prepared samples for STEM observations via post-annealing of the polycrystals at 1400, 1500, and 1600 °C, respectively, in order to systematically track the structural variations at the RP faults with temperature (see Supplementary Fig. 3 for X-ray diffraction patterns and optical micrographs indicating the polycrystalline dense samples). Figure 3a shows representative HAADF and BF images for the formation of disordered layers by premelting at the fault planes inside a grain after post-annealing. This series of images directly demonstrates that melting is initiated inside a bulk crystal, not merely at grain boundaries and crystal surfaces below the melting point. Once such disordering via breaking interatomic bonds takes place in a certain location, atomic displacement for liquefaction in an adjacent neighboring region can be easily achieved by vibrational instability at a slightly higher temperature than the temperature at which premelting is initiated but that is still lower than the bulk melting point. The melt front can thus propagate into the crystal, resulting in more than 5 nm thickness, as shown in the sample annealed at 1600 °C. Because these thin amorphous layers are mechanically fragile, cracks were frequently observed on the fractured surfaces of the post-annealed samples during the scanning electron microscopy (SEM) analysis, as demonstrated in Fig. 3b−f. Note that the RP faults are always along the {100} plane (see Fig. 1f, g). Therefore, as indicated by white arrows in each of the SEM images in Fig. 3d−f, the cracks should be either parallel or perpendicular to each other.

**Fig. 4:**
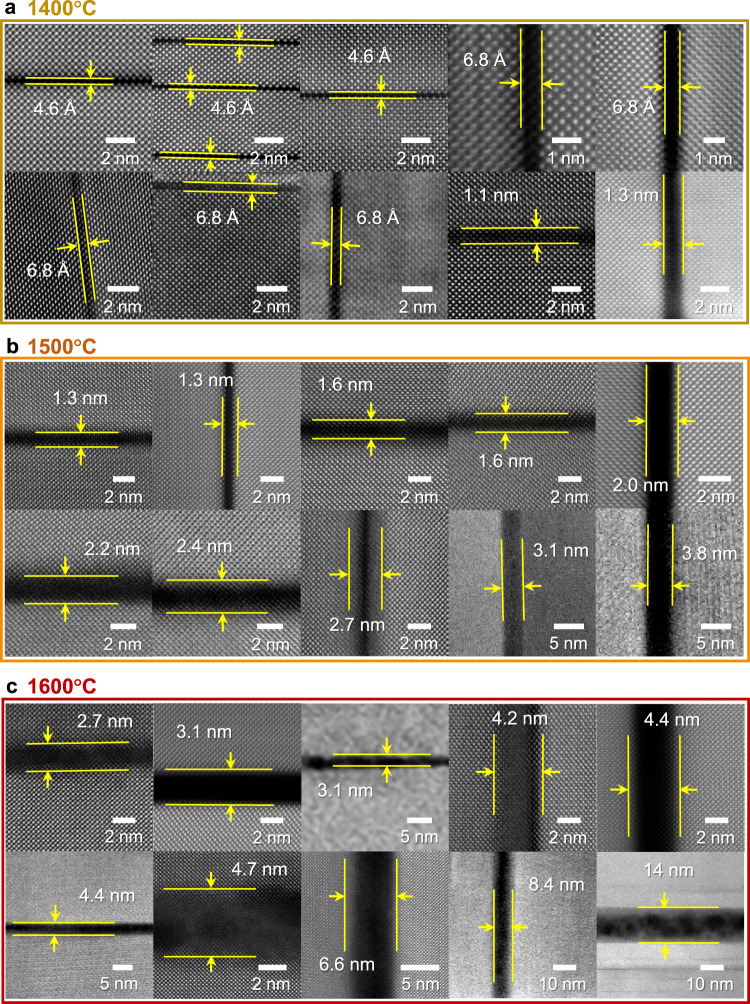
Three sets of HAADF images for the premelted fault regions at different temperatures. **a−c** More than 10 grains in the [100] projection were observed in each case of post-annealing at 1400 (**a**), 1500 (**b**), and 1600 °C (**c**). It is noted that the thickness of the premelted amorphous layers is not completely constant at each annealing temperature but somewhat varies from fault to fault.

**Fig. 5:**
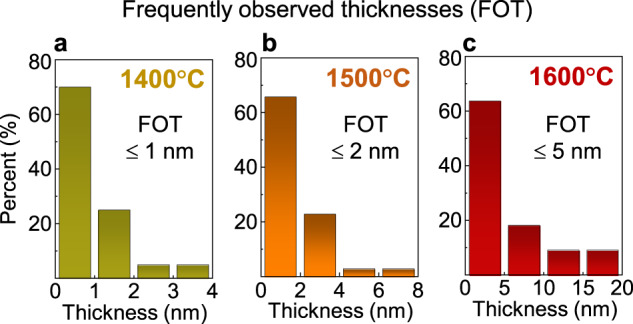
Thickness distributions of premelted amorphous layers. **a−c** These bar graphs present the thickness distribution of amorphous layers in samples post-annealed at 1400 (**a**), 1500 (**b**), and 1600 °C (**c**). The amorphous layers become thicker, as the annealing temperature increases from 1400 to 1600 °C. Frequently observed thicknesses of amorphous layers in each graph are represented as the thicknesses observed at more than 60% of grain boundaries.

We observed more than 10 grains in the [100] projection in each polycrystalline sample for statistical information on the thickness variation of a disordered amorphous layer. Figure 4a−c present 10 typical HAADF-STEM images for each sample annealed at different temperatures. The feature in this series of observations is that the thickness of amorphous layers is distributed over a range rather than having a single value at each annealing temperature, showing a somewhat widely distributed range of thickness. For example, we identified amorphous layers with >1 nm thickness in addition to the smallest 4.6 Å-thick monolayer in the sample annealed at 1400 °C in Fig. 4a (see Supplementary Fig. 4 for the definition of the thickness of an amorphous layer in HAADF images). When we scrutinized the chemical composition with atomic-scale EDS, we detected a degree of intermixing of Ce in some Ba sites at the faults, as directly demonstrated in the atomic-column-resolved EDS maps in Supplementary Fig. 5. This locally different Ba/Ce stoichiometry is very likely to result in fault-to-fault variation of melting behavior rather than identical characteristics among the faults. As a consequence, premelting with different thicknesses in our samples is frequently observed. To clarify the thickness distribution at each annealing temperature, bar graphs of the distribution are provided in Fig. 5a−c and they demonstrate that disordered amorphous layers overall become thicker as the annealing temperature increases.

**Fig. 6:**
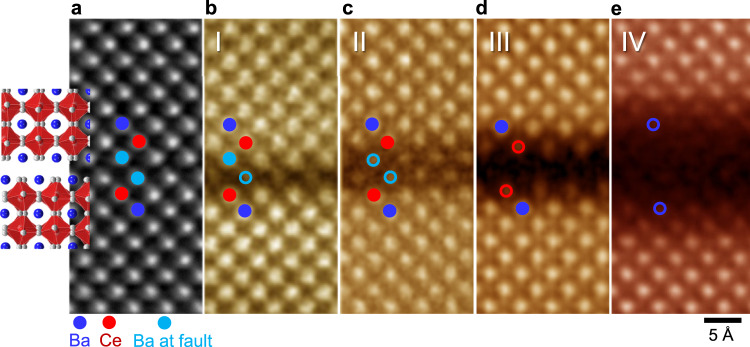
Layerwise premelting at the fault. **a** An atomic illustration is provided in the first STEM image to verify the presence of a fault. **b−d** Images I−III (1400 °C) directly show the layer-by-layer behavior at the initial stage of premelting. Open circles represent the layers contacting atomic columns with low intensity due to significant atomic displacement by melting. **e** In image IV (1500 °C), melting of multiple atomic layers (more than four layers) is identified along with diffuse interfaces between the bulk crystal and the melted layer.

The layerwise behavior of premelting at 1400 °C rather than random disordering was also captured as another feature during STEM observation. Figure 6a−e show a series of atomic-scale HAADF images acquired in the samples annealed at 1400 °C (images I, II, and III) and 1500 °C (image IV) together with an illustration showing the atomic arrangement at the fault plane. While the Ba columns in the upper [BaO] layer at the fault plane are clearly visualized in image I (sky-blue closed circle), a significant loss of column contrast is identified in the lower [BaO] layer (sky-blue open circle). The observation of premelting at a 2D fault consistently agrees with our expectation based on the Ba−O bonding characteristics, as shown in Fig. 2c. However, it is the intriguing finding from Fig. 6b that melting can begin with only one single [BaO] layer out of two identical [BaO]−[BaO] layers at the fault plane (see Supplementary Figs. 6 and 7 for additional STEM images verifying the melting of a single [BaO] layer). The melting of two [BaO] layers at the same time was also identified in image II (Fig. 6c), as denoted by a pair of sky-blue circles, supporting again that the RP faults are preferential defect sites for premelting.

Further melting proceeds in a layerwise manner as well. Image III in Fig. 6d reveals a significantly low column contrast of both the upper and lower neighboring [CeO_2_] layers (red open circles). This directly indicates that the [CeO_2_] layers begin to melt in addition to the complete melting of the [BaO]−[BaO] layers. However, when melting proceeds further and thereby the disordered liquid layers become thicker, the distinct layer-by-layer melting behavior was no longer clearly observable. As can be seen in Image IV (Fig. 6e), fairly diffuse boundaries at an atomic level are identified between the high-contrast crystalline region and the low-contrast amorphous layers. An additional series of HAADF images with a wide field of view is provided in Supplementary Fig. 8 to clarify the layerwise melting.

### STEM image simulations

**Fig. 7:**
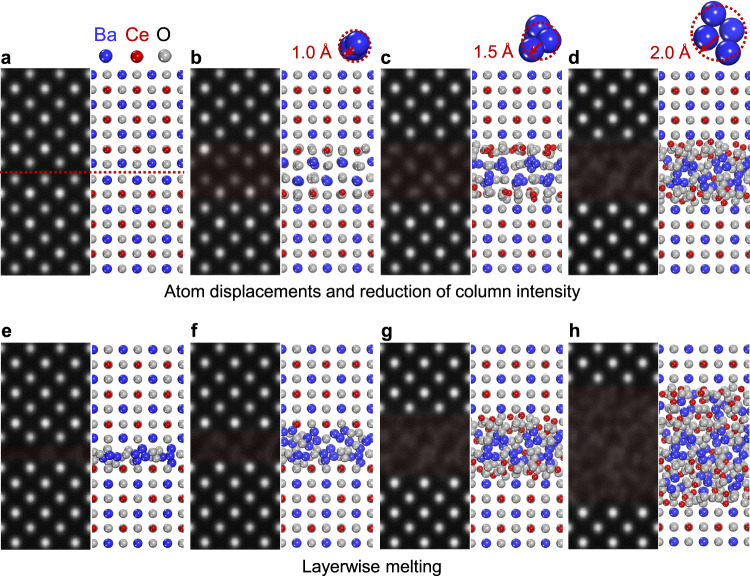
STEM image simulations. **a−d** This series of simulated images and corresponding supercells demonstrates how the intensity of atom columns fades and nearly disappears, when the average atom displacement is 0 (**a**), 1.0 (**b**), 1.5 (**c**), and 2 Å (**d**). In the final image **d**, no distinct atomic columns are imaged in the layer where atoms substantially displace from their initial positions. **e−h** This series of simulated images shows how the atomic-column contrast in STEM images varies when melting occurs in a layer-by-layer manner: A [BaO] single layer (**e**), consecutive [BaO]−[BaO] layers (**f**), two [BaO]−[CeO_2_] layers (**g**), and four [BaO]–[CeO_2_] layers (**h**). In good agreement with the experimentally obtained STEM images shown in Fig. 6, these simulations consistently support layerwise premelting at the RP faults.

The channeling effect of an electron beam through each atomic column in STEM is significantly reduced when the periodic array of through-column atoms is perturbed by melting. As a consequence, a remarkably low column contrast (or disappearance of column contrast) is readily observable in Figs. 3a and 6. To examine the column contrast variation by a different degree of atomic displacement, we constructed supercells respectively containing a fault and carried out HAADF-STEM image simulations based on the multislice method^40,41^. As shown in Fig. 7a−d, a series of simulated STEM images demonstrates that the column intensity substantially diminishes, as the average displacements of atoms from their initial positions increase up to 1.5 Å. When the displacements are more significant (~2.0 Å) in the final case (Fig. 7d), atomic columns in these layers are no longer imaged. Therefore, the near complete absence of substantial column intensity at the premelted faults in the HAADF images stems from the significant displacement of atoms, in close agreement well with the real images acquired from the annealed samples. The layer-by-layer disappearance of atomic-column contrast by melting was also verified during the image simulation. Figure 7e−h present a series of simulated images to demonstrate consecutive melting of the lower [BaO] layer (Fig. 7e), the upper [BaO] layer (Fig. 7f), both neighboring [CeO_2_] layers (Fig. 7g) and further [BaO]−[CeO_2_] layers (Fig. 7h). Showing good agreement with the real images in Fig. 6, the modeling in Fig. 7e−h thus consistently supports the layerwise behavior of melting.

### Observation of recrystallization

**Fig. 8:**
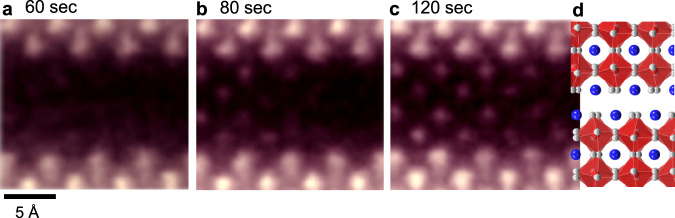
Recrystallization of a premelted amorphous layer. **a−c** These HAADF images were captured from the video clip (Supplementary Movie 1) recorded during e-beam irradiation at 60 (**a**), 80 (**b**), and 120 s (**c**) in STEM. The regeneration of a periodic array of atomic columns is visualized, directly indicating the recrystallization. **d** An atomic array illustration is also provided to indicate the presence of a fault.

The disordered liquid layers at the fault planes are amorphous after quenching to room temperature (see Supplementary Figs. 9 and 10 verifying the amorphous state and the composition of Ba and Ce). Therefore, if sufficient energy is provided to overcome the energy barrier for crystallization, the original perovskite framework should be recovered. As the convergent electron beam in our STEM is accelerated at a high voltage, 300 kV, we utilized an electron irradiation method^42^ to induce crystallization during STEM observation. Figure 8a−c show a series of HAADF images captured in real-time. The image in Fig. 8a reveals that an ordered configuration of atomic columns begins to appear after approximately one minute of irradiation. For dynamic information, a video clip showing this local crystallization is provided in Supplementary Movie 1. Once crystallization is triggered, a further transition into a crystalline state is observed in Fig. 8b, c. As indicated by the illustration of the atomic arrangement in Fig. 8d, the recurrence of the RP fault, consisting of two consecutive [BaO] layers, is identified as well, demonstrating recrystallization to the initial state before premelting. Another notable aspect in this series of images is that the intensity of columns in the amorphous region during recrystallization is much lower than that of columns in the crystalline region. It is noted that the position of atoms still fluctuates during recrystallization and thereby the through-column channeling of incident electrons is not as significant as that in the crystalline region. As shown in the image simulations in Fig. 7a−d, this reduced channeling thus can contribute to lowering the column intensity. In addition, because a convergent electron beam at a high acceleration voltage, 300 kV, was used for a fairly long period of time to record a video clip, it is anticipated that electron-beam sputtering will preferentially occur in the premelted amorphous region. Therefore, the reduction of column intensity can be affected by the substantial mass loss as well.

## Discussion

In 1910, Lindemann proposed that melting is triggered by a vibrational instability in a crystal when the root-mean-square displacement of atoms, 〈Δ*r*^2^〉^1/2^, reaches a certain critical fraction (10−15%) of the lattice constant^13^. As the vibrational amplitude of atoms at the surface should be much more substantial than that of the bulk atoms, surface melting prior to the bulk melting point was suggested. On the other hand, in 1939 Born proposed a distinct model of melting in terms of a rigidity catastrophe caused by the shear modulus vanishing^20^. In a milestone work in 1946, Frenkel further specified the necessity of intrinsic defects, encompassing vacancies and interstitials, for melting in addition to the Born criterion based on the elastic lattice instability^14^. Through a combination of ultrafast electron diffraction technique^43^ and molecular dynamics (MD) simulations, a recent study^19^ indeed showed that radiation-driven vacancy clusters in tungsten films can act as preferential sites for the melting transition below the melting point in accordance with Frenkel’s suggestion. In addition, using Lennard−Jones particles in an FCC box with MD simulations, Jin et al. in 2001 discovered that the Lindemann and Born criteria both lead to the same conclusion for the mechanism of melting when applied to the bulk crystal instead of the surface^44,45^. The report by Jin et al. has an important implication, as it clarifies that the accumulation of internal lattice instability constitutes the primary mechanism for melt nucleation inside the crystal. In this respect, we believe that the STEM images for BaCeO_3_ in Figs. 3 and 6 offer direct experimental evidence in an inorganic crystal supporting the lattice-defect-induced premelting phenomena inside the crystal. Furthermore, the initially layerwise premelting at the 2D RP faults with comparatively weak bond strength (see Fig. 2c) directly supports that lattice defects are the exact origins of the vibrational and elastic lattice instability, as suggested in both the Lindemann and Born criteria.

Although the present study focuses on premelting initiated inside a crystal, it is worthwhile to compare our observations with previous results regarding grain-boundary complexions, which encompass various transitions of faceting, order−disorder, wetting, premelting, and adsorption at grain boundaries as a general concept^46–48^. For a direct comparison, we carried out additional STEM analyses on more than 50-grain boundaries in a polycrystalline sample annealed at 1400 °C. As can be seen in Supplementary Fig. 11, the presence of an amorphous layer was readily identified at grain boundaries, showing that premelting occurs at grain boundaries as well. In particular, the amorphous layers at grain boundaries are much thicker on average than those observed at the RP faults within the crystals. As the atomic thermal vibration for premelting is limited within a crystal, the much narrower premelted layers observed at the faults are consistent with both the Lindemann and Born criteria. Indeed, the set of HAADF-STEM images, which were taken before and after premelting, in Supplementary Fig. 12 directly demonstrates the presence of a thicker amorphous layer at a grain boundary, while single-atomic-layer melting is shown at an RP fault within a grain. Additional phase-contrast high-resolution electron microscopy images were also provided in Supplementary Fig. 13 to confirm the existence of an amorphous layer (>5 nm in thickness) on the crystal surface.

Another issue to carefully consider is the effect of nonstoichiometry between A and B in ABO_3_-type perovskite oxides on the formation of an amorphous layer at grain boundaries. As demonstrated in SrTiO_3_, a small deviation from the complete stoichiometry of Sr:Ti = 1:1 results in the formation of a secondary liquid phase at high temperature^49^ (see the gray-shadow two-phase regions in the phase diagram of SrTiO_3_ in Supplementary Fig. 14a). This liquid phase can frequently wet the grain boundaries in polycrystalline SrTiO_3_, as shown in previous studies^50–52^. It is thus noted that, if substantial cation nonstoichiometry is present, the grain-boundary premelting cannot be distinguished from the nonstoichiometry-induced liquid formation at grain boundaries in many perovskite oxides. In stark contrast, premelting initiated in the crystal interior is an independent phenomenon and is not affected by any secondary phases originating from nonstoichiometry. The phase diagram of BaCeO_3_ is also provided in Supplementary Fig. 14b for reference, indicating the existence of similar two-phase regions.

Finally, we note that experimental direct observations often play a critical role in both validating theoretically suggested physical phenomena and elucidating details that cannot be entirely covered by predictions. Recent observations on the 2D Wigner electron crystal^53,54^ (initially predicted by Wigner in 1934 (ref. ^55^) in WSe_2_/WS_2_ moiré heterostructures; atomic-scale visualization of vortex-type ferroelectric nanodomains^56,57^ (suggested by Kornev et al. in 2004 (ref. ^58^)) in Pb(Zr,Ti)O_3_ and BiFeO_3_ thin films; and in-situ imaging of a multi-phase transformation^59,60^ (empirically proposed by Ostwald in 1897 (ref. ^61^) and thus dubbed the Ostwald rule of stage^62,63^) during crystallization of amorphous LiFePO_4_ are noteworthy examples emphasizing the significance of direct observation in the relevant fields. The STEM imaging in the present study is another impactful result that proves valuable classical predictions that have yet to be atomic-scale direct evidence in crystals.

We have provided the atomistic details on premelting initiated at the lattice defects of 2D homologous faults inside BaCeO_3_ crystals as a model system. In addition to identifying the thickness increment of the disordered liquid layers with annealing temperature, we successfully captured atomic-level images demonstrating the layer-by-layer behavior at the initial stage of melting. The experimental findings in this study clarify the significance of lattice defects present inside a crystal as major sources of the vibrational and elastic instability originally proposed by the Lindemann and Born criteria.

## Methods

### Preparation of polycrystalline samples

To fabricate BaCeO_3_ polycrystals, powders were synthesized via a conventional solid-state reaction method by using BaCO_3_ (99.999%, Aldrich) and CeO_2_ (99.95%, Aldrich). A small amount (5%) of an acceptor-type dopant, Dy_2_O_3_ (99.9%, Aldrich), was also added to facilitate densification during sintering. A stoichiometric mixture of the starting materials was ball-milled in high-purity alcohol for 24 h with a zirconia jar and balls. After being dried, the slurry was calcined at 1050 °C for 12 h in the air for solid-state synthesis. The calcined powder was ball-milled again to obtain fine particles. The synthesized powder was slightly pressed into disks and isostatically pressed under 200 MPa. The disk-type pellets were sintered at 1330 °C for 5 h in the air to verify that no premelting occurs. For the post-annealing process, the sintered samples without the acceptor dopant were annealed again at 1400, 1500, and 1600 °C for 5 h, respectively, and rapidly removed from the furnace at a cooling rate of 300 deg/min to room temperature. Pellets with the dopant were sintered at 1400 and 1500 °C to observe grain-boundary premelting and measure the thickness distribution of an intergranular amorphous phase at gran boundaries as well. To examine the crystal surface, particles synthesized by the solid-state reaction were annealed at 1400 °C for 3 h.

### SEM, STEM, HREM, EDS and EELS

The cross-sectional microstructure of fractured samples in addition to the overall polycrystalline microstructure was observed by scanning electron microscopy (Magellan400, Thermo Fisher Scientific). Samples for STEM observation were prepared by mechanical grinding to a thickness of 60 μm, dimpling to a thickness of <10 μm and finally ion-beam thinning by using a precision ion polishing system (PIPS, Gatan Inc.) for electron transparency. STEM images were acquired with a transmission electron microscope (Titan cubed G2 60-300, Thermo Fisher Scientific) at 300 kV with a spherical aberration (Cs) corrector (CEOS GmbH). The optimum size of the electron probe was ~1 Å with a convergence semiangle of 19 mrad. The collection semiangles of the STEM detectors were set to 50.5−200 mrad for HAADF imaging and 0–27.5 mrad for BF imaging. Another Cs-corrected transmission electron microscope (ARM200F, JEOL) was used at 200 kV. For phase-contrast HREM imaging of the particle surfaces, a conventional transmission electron microscope (Tecnai F20, Thermo Fisher Scientific) was also utilized at 200 kV. Chemical composition mapping with EDS was performed in the Titan cubed G2 at 300 kV along with four integrated silicon-drift EDS detectors (ChemiSTEM™ technology) at a collection solid angle of 0.7 srad. The EDS maps were low-pass filtered using Bruker Esprit software after the reduction of background noise for better visualization. It is noted that Ce-L_α_ peak overlaps with the Ba-L_β1_ peak in EDS. Therefore, the Ba-L_α_ and Ce-L_β1,2_ peaks were selected for atomic-column-resolved composition mapping by EDS. EELS analysis was performed with a Gatan Image Filter (GIF Quantum 965, Gatan Inc.). Electron energy-loss spectra for the Ba-M_5_ (781 eV) and Ce-M_5_ (883 eV) edges were acquired for atomic-column spectrum imaging of BaCeO_3_ with a dispersion of 0.25 eV per channel and a collection aperture of 5 mm in diameter. To record a video clip during e-beam irradiation for recrystallization, the beam current was adjusted to be 100−150 pA with a pixel dwell time of 3 μs and thus the electron dose was estimated to be 2.8−4.2 × 10^5^ electrons/Å^2^ s (equivalently 2.8−4.2 × 10^7^ electrons/nm^2^ s) in our conditions.

### STEM image simulations

STEM images were simulated by using Dr. Probe software^64^ based on the multislice method. A beam energy of 300 kV, spherical aberration coefficients of *C*_s_ = 0 mm, *C*_5_ = 0 mm, and *C*_7_ = 0 mm without coma and astigmatism, an electron probe size (FWHM) of 1 Å, and a slice thickness of 2 Å were set during the simulations. Each of the real collection semiangles of the STEM detectors (50.5−200 mrad for HAADF imaging and 0–27.5 mrad for BF imaging) was also used for precise comparison with experimentally acquired images.

## Supplementary information

Supplementary Information

Description of Additional Supplementary Files

Supplementary Movie

## Data Availability

The datasets generated during and/or analyzed during the current study are available from the corresponding author on reasonable request.
