# Supplementary material for: Atomic-scale observation of premelting at 2D lattice defects inside oxide crystals
Source: Nat Commun. 2023 Apr 20;14:2255. doi: 10.1038/s41467-023-37977-w (PMC10119109; doi:10.1038/s41467-023-37977-w)
Supplement: Supplementary file 1 — Supplementary Information [file 41467_2023_37977_MOESM1_ESM.pdf]

Supplementary Information

Atomic-scale observation of premelting at 2D lattice defects inside oxide crystals

Kim and An et al.

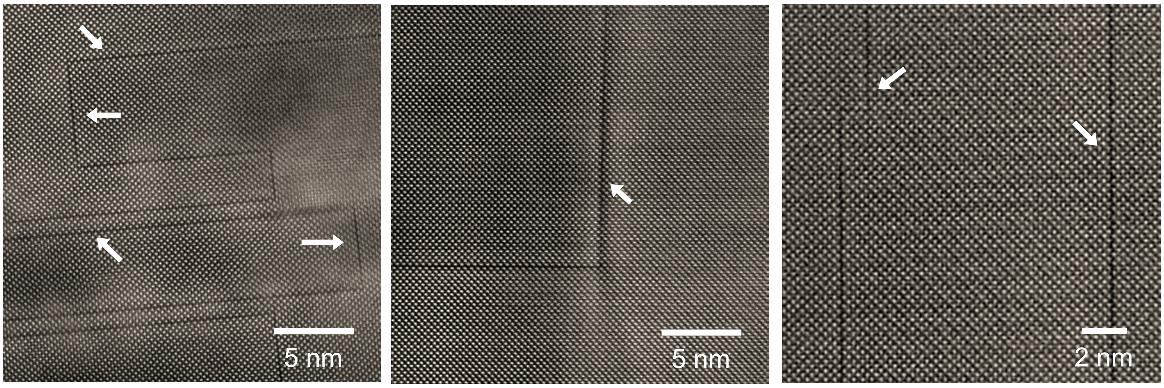

Supplementary Fig. 1 Additional sets of HAADF-STEM images of the RP faults in $BaCeO_3$ polycrystals. As indicated by white arrows in each image, a line contrast for the planar fault is easily observable. Because the fault plane is parallel to the [100] direction in the pseudocubic perovskite framework, the fault lines should be either perpendicular or parallel to each other.

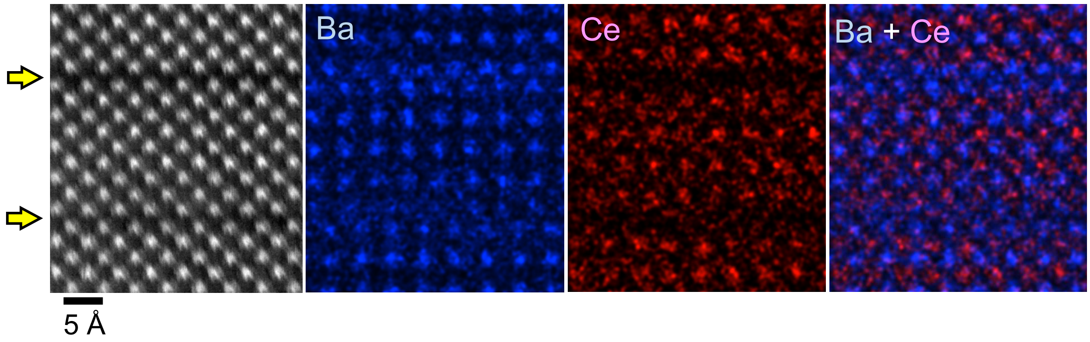

Supplementary Fig. 2 Additional set of EDS maps. This atomic-column-resolved EDS mapping consistently demonstrates the two consecutive Ba–Ba columns at the fault planes denoted by yellow arrows.

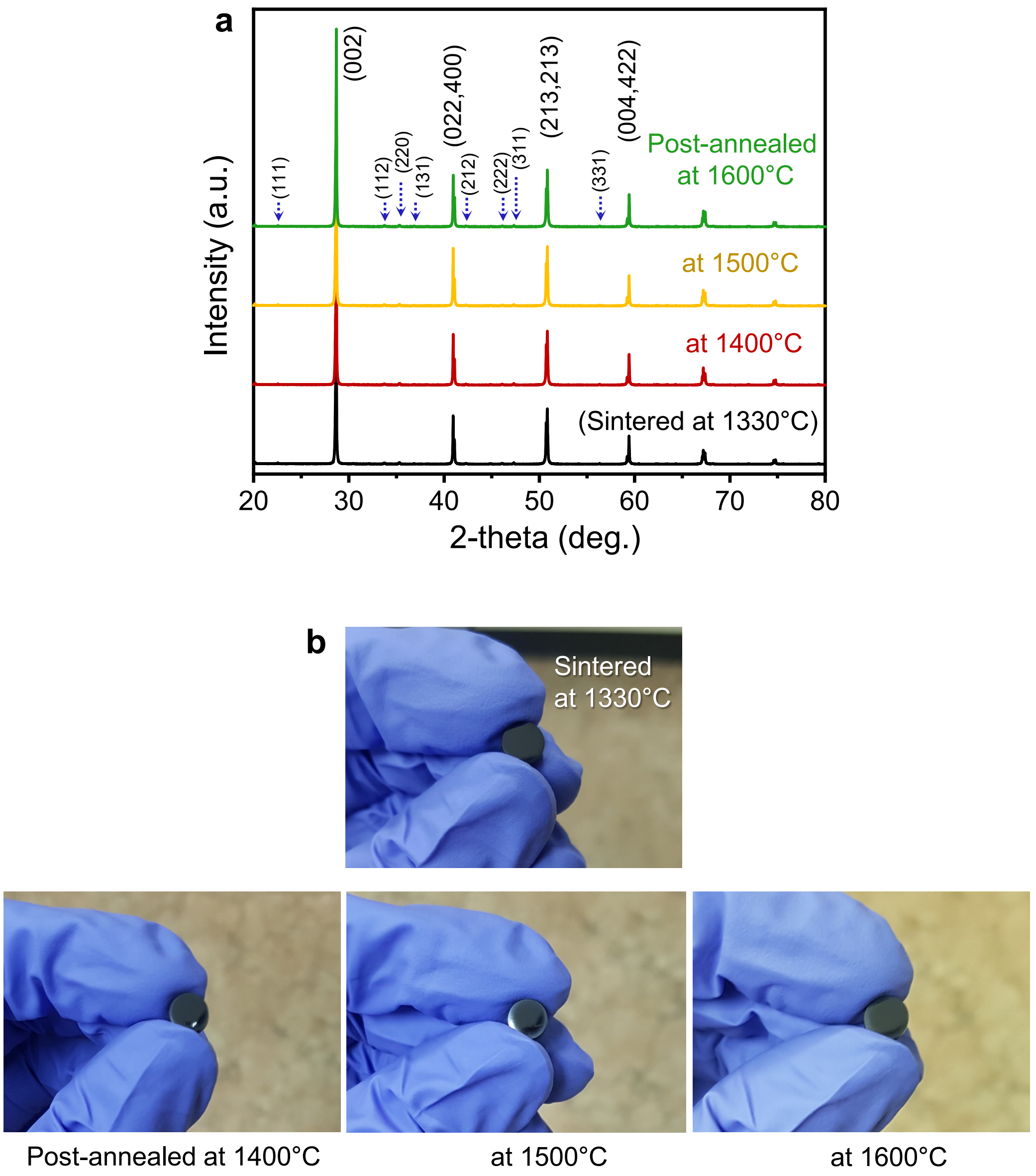

Supplementary Fig. 3 X-ray diffraction patterns and shape preservation of disc-type specimens. (**a**) X-ray diffractometry was carried out to verify that each specimen remains polycrystalline after post-annealing at 1400–1600°C, which is below the bulk melting temperature. All the Bragg reflections are identified to consistently show relative peak intensities in each specimen with no broad background peak, thereby indicating that the specimens remain polycrystalline without bulk melting after post-annealing. (**b**) The disc-type shape of the sintered specimens is also preserved after post-annealing without change of their initial morphology.

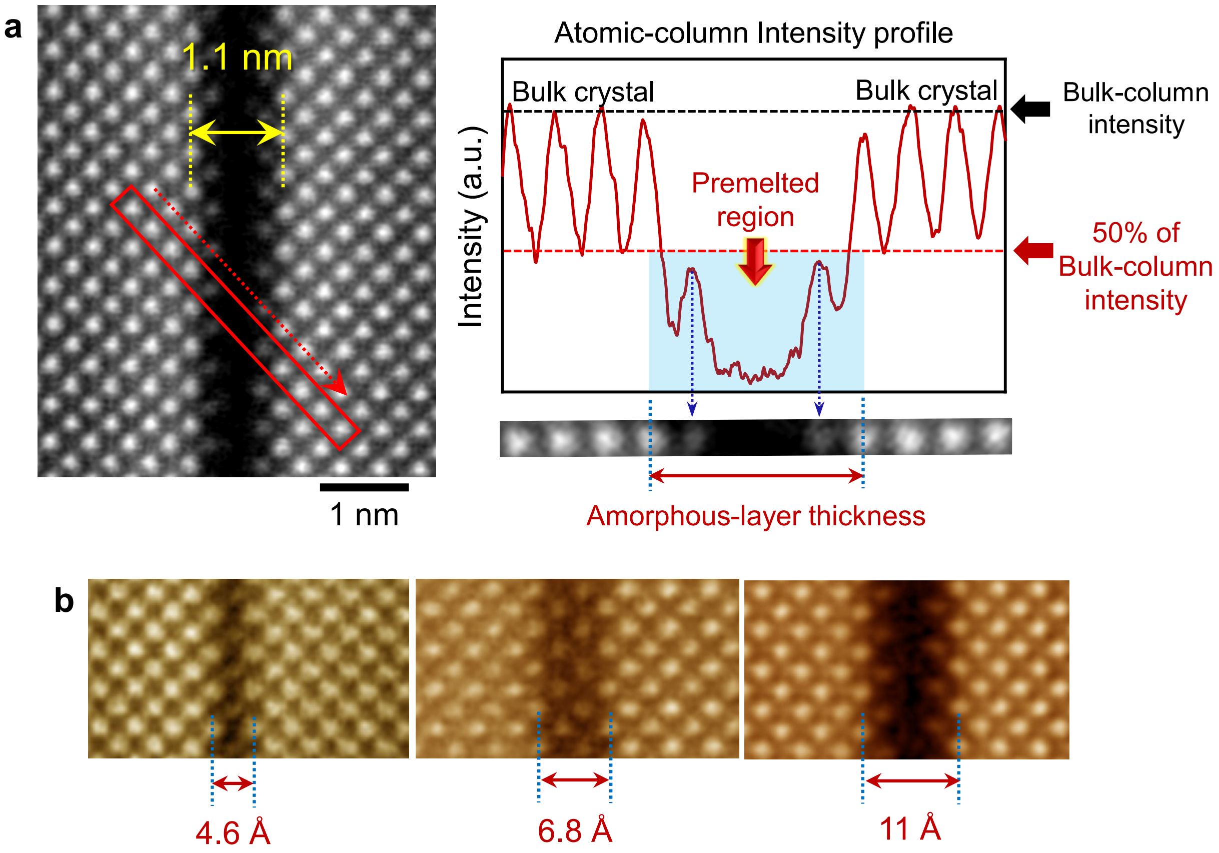

Supplementary Fig. 4 Determination of amorphous layer thickness. As already addressed in the main text, the intensity reduction of atomic columns in the amorphous layer in HAADF images stems from the low through-column channeling effect of electrons due to the loss of crystallinity. Consequently, even though the atomic columns with low intensity remain visible, many atoms through the columns significantly displace from the original positions. (**a**) An intensity profile is shown on the right-hand side for the atomic columns denoted by a red rectangle in the HAADF image. As demonstrated in this profile, we set the thickness of an amorphous layer so as to encompass all of the low-intensity (less than 75% of the bulk-column intensity) columns. (**b**) This criterion for thickness determination is also applicable even to the case of [BaO] monolayer melting. As shown in the first image, the monolayer-melt thickness is determined to be 4.6 Å. For the two-layer melt in the second image, the thickness is identified to be 6.8 Å. The last images exemplifies the melting of four layers with cumulative of 11 Å. The information on the melt thickness denoted in the images in Fig. 4 in the main text is based on this determination criterion.

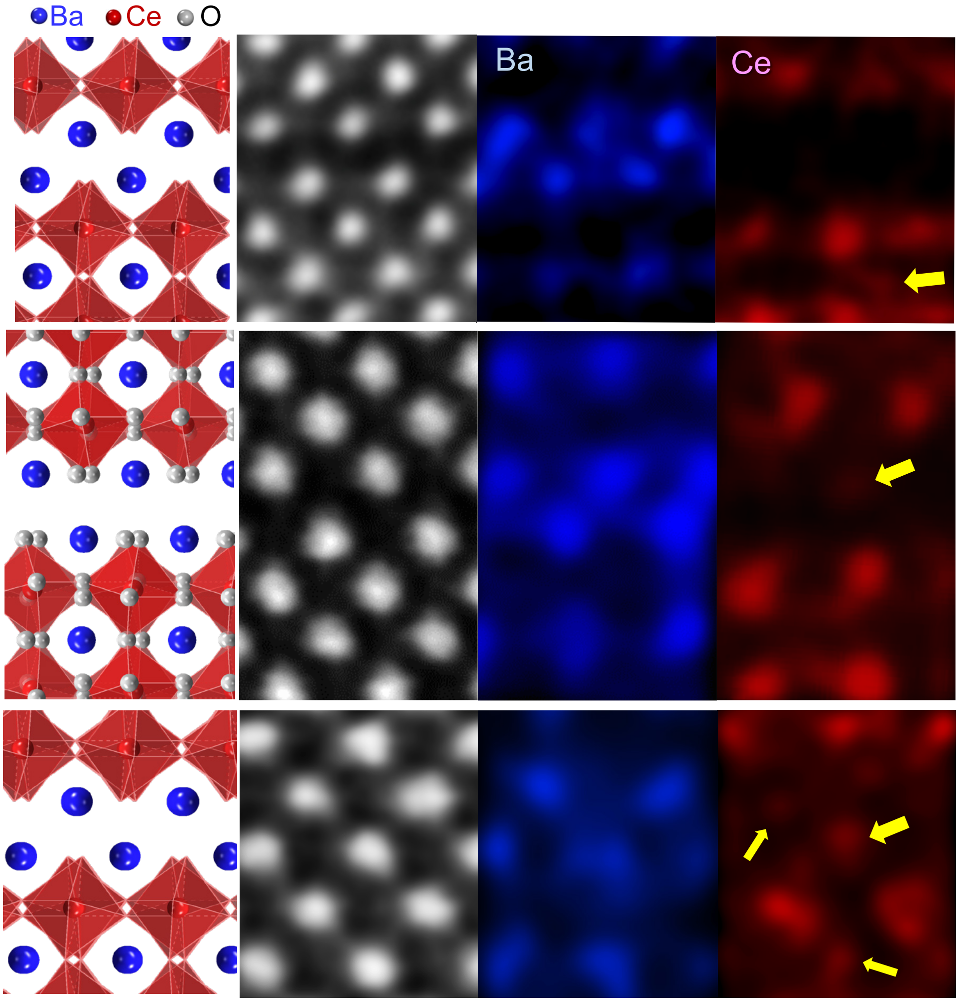

Supplementary Fig. 5 Additional sets of atomic-column-resolved EDS maps. Although the major composition of the Ba and Ce columns, respectively, does not vary at the fault planes, a small degree of Ce disordering in some Ba columns is clearly detected, as indicated by yellow arrows.

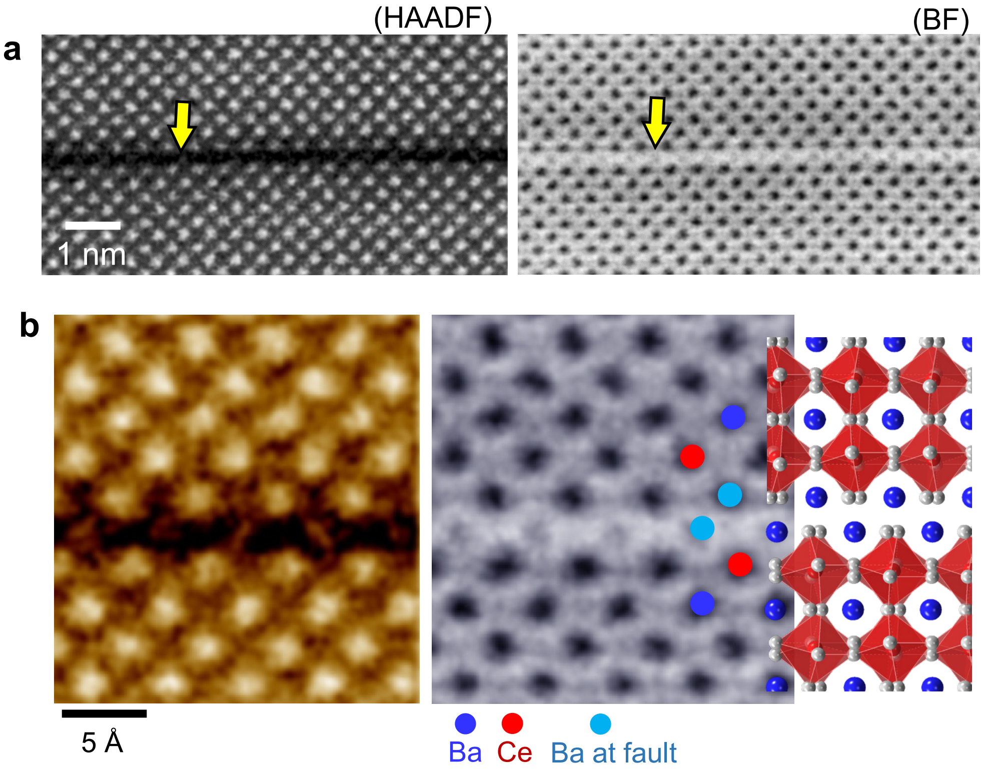

Supplementary Fig. 6 Additional STEM images for melting of a single [BaO] layer. (**a**) The loss of column contrast indicated by yellow arrows in each of the HAADF and BF images, showing the melting of a [BaO] layer. (**b**) The magnified images are provided to clarify the melting of the lower layer from the two consecutive [BaO] layers at the fault.

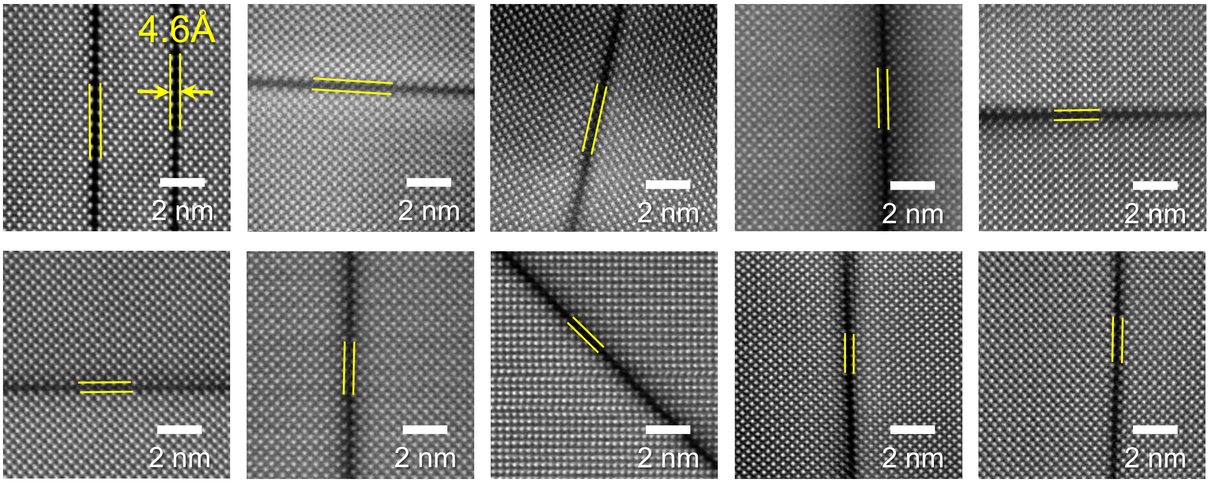

Supplementary Fig. 7 Additional STEM images for melting of a single [BaO] layer. HAADF images showing the single-layer melting were acquired from the sample post-annealed at 1400°C. As denoted by a pair of yellow lines in each image, the thickness of 4.6 Å is consistently identified.

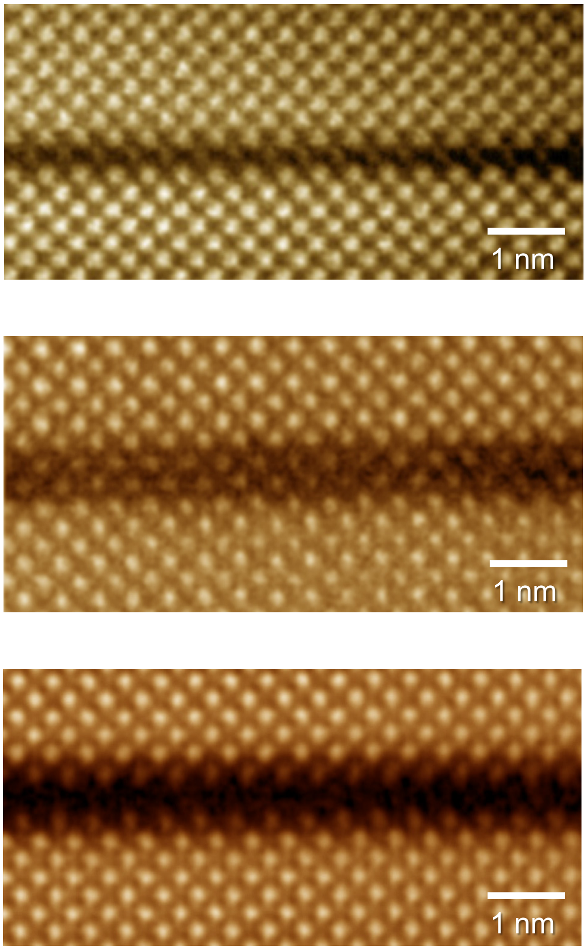

Supplementary Fig. 8 Additional STEM images for a wide field of view. This series of images with a wide field of view is provide for Fig. 6 in the main text to clarify the layerwise behavior of premelting at an early stage at 1400°C.

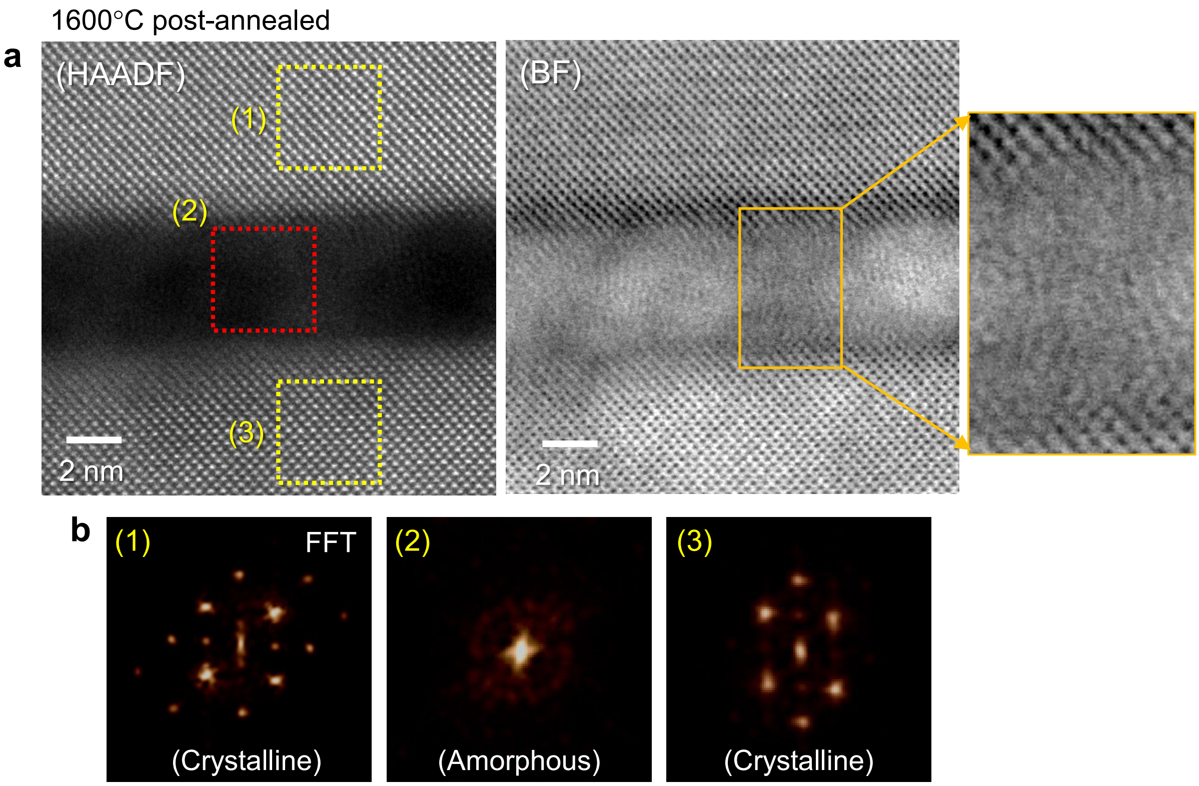

Supplementary Fig. 9 Additional set of STEM images for a disordered amorphous layer by premelting at 1600˚C. (**a**) A pair of HAADF and BF images is shown to verify the amorphous state. In particular, the magnified BF image clearly demonstrates the typical feature of the loss of long-range crystalline order. (**b**) Fast Fourier transform (FFTs) for the regions denoted by (1), (2), and (3) in the HAADF images in (a) are shown, consistently confirming that region (2) is amorphous.

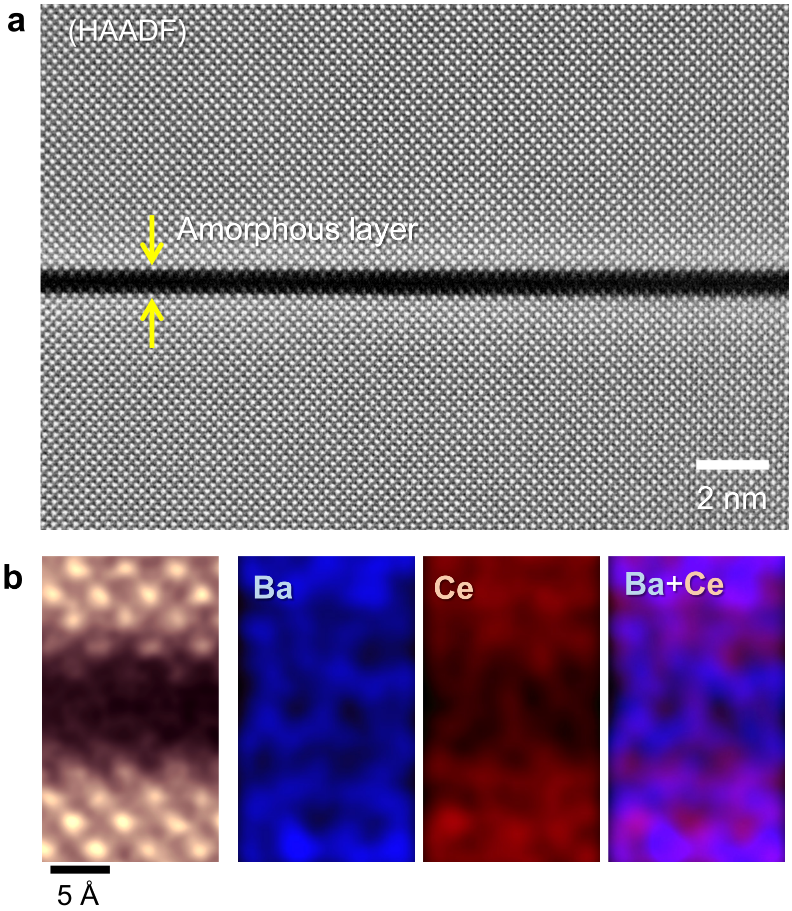

Supplementary Fig. 10 Additional STEM image and EDS maps. Before carrying out recrystallization by using e-beam irradiaint in STEM, the amorphous state of a premelted layer and its chemical composition were verified. (**a**) This HAADF image provides a wide field of view for the amorphous layer for confirmation. (**b**) While the amorphous layer does not exhibit any significant atomic columns, due to the loss of crystallinity, the chemical composition of Ba and Ce is clearly identified by EDS mapping.

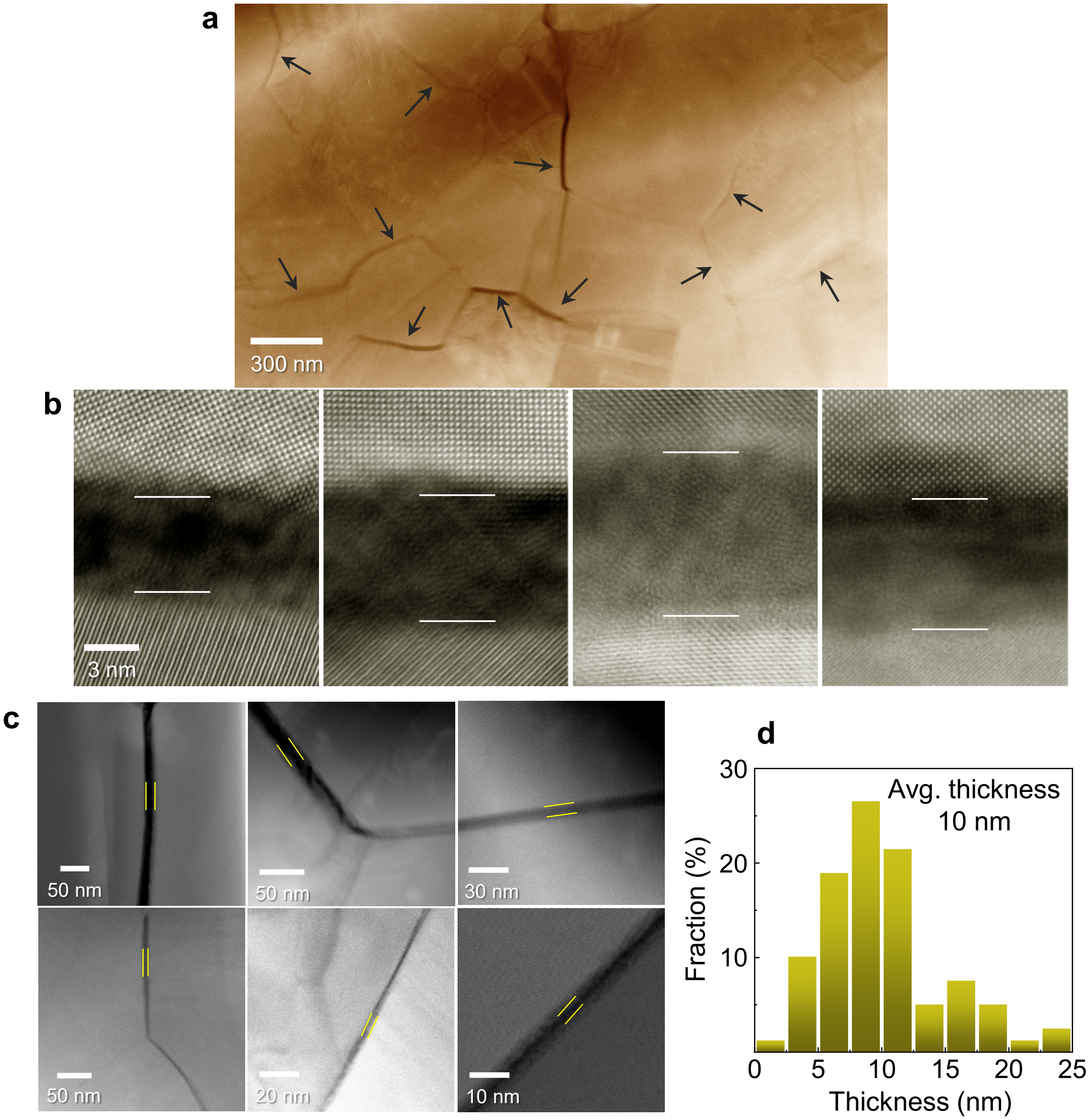

Supplementary Fig. 11 Nanoscale intergranular amorphous layers at grain boundaries. (**a**) This HAADF-STEM image obtained from sintered polycrystals at 1400°C demonstrates the typical polycrystalline microstructure. The presence of intergranular amorphous layers at most of the grain boundaries is recognizable, as indicated by arrows. (**b**) Atomic-scale HAADF images also clarify the presence of fairly thick amorphous layers by premelting at grain boundaries. (**c**) As exemplified in these additional images, more than 50 grain boundaries were examined to acquire statistical information on the thickness distribution. (**d**) The bar graph represents the thickness distribution of amorphous layers at grain boundaries, showing an average value of ~ 10 nm.

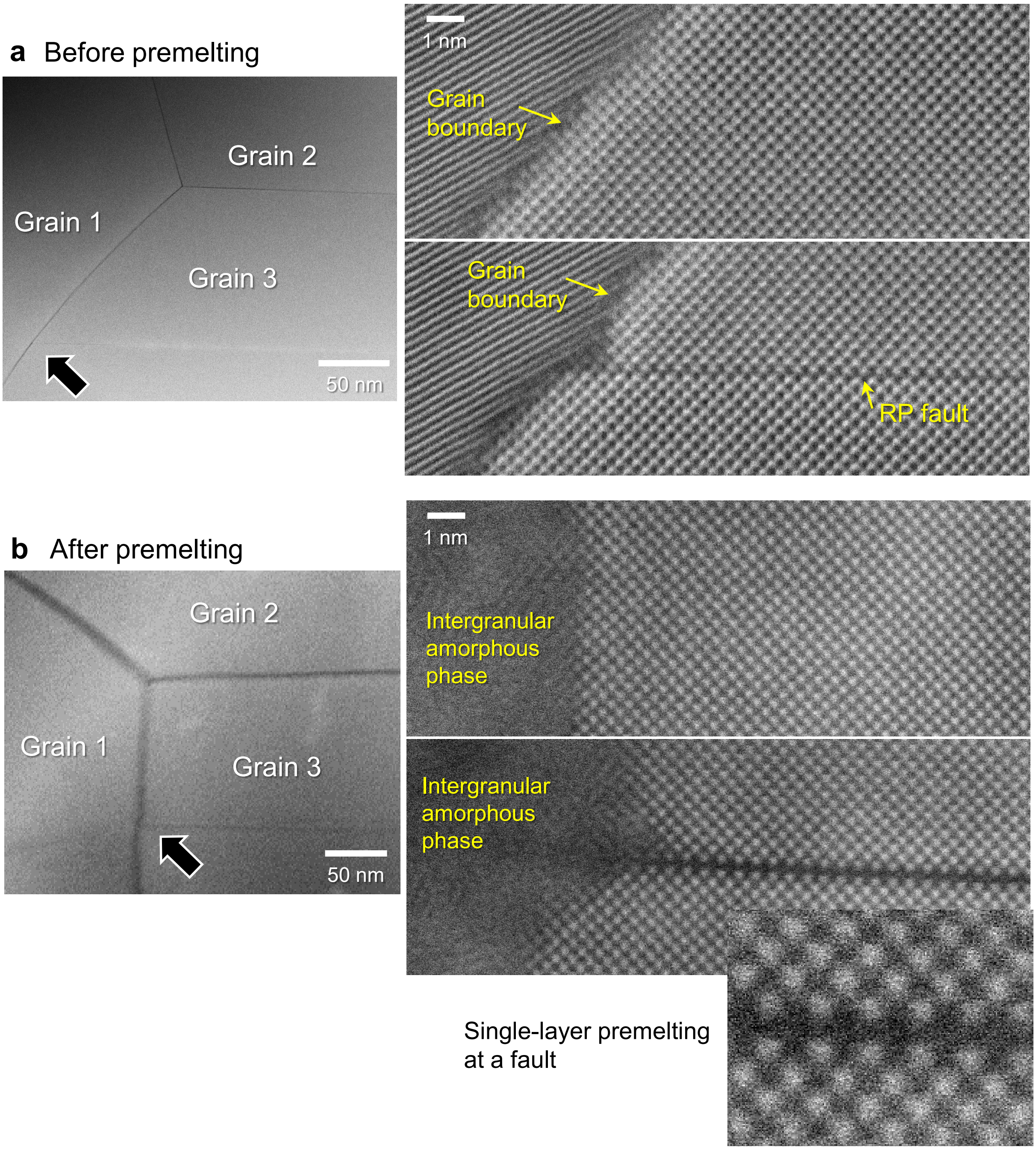

Supplementary Fig. 12 Additional STEM images and local enlargements before and after premelting. (**a**) Grain boundaries and a RP fault in a sample sintered a 1330°C were imaged at one time. No premelting is observed at either grain boundaries or a fault inside a grain. (**b**) Premelting occurs at all three grain boundaries at 1400°C. In addition, single-layer premelting at the fault plane is also identified in a magnified image on the right-hand column. Note that the amorphous layers at grain boundaries are much thicker that the melted monolayer at the fault.

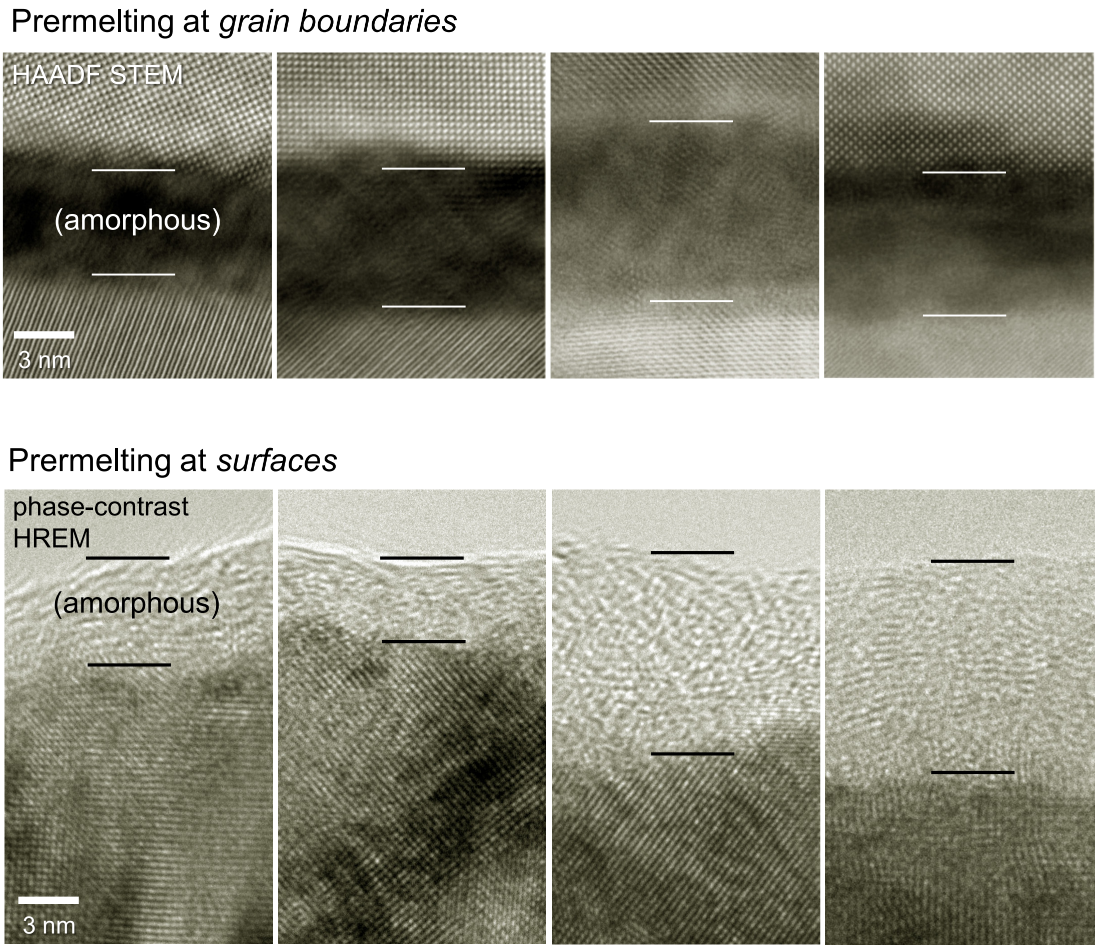

Supplementary Fig. 13 Premelting at grain boundaries and particle surfaces. The STEM images shown for premelting at grain boundaries in Supplementary Fig. S11 are displayed again for comparison with premelting at crystal surfaces at the same temperature, 1400°C. For better contrast of an amorphous layer on the crystal surfaces, phase-contrast HREM was used instead of STEM.

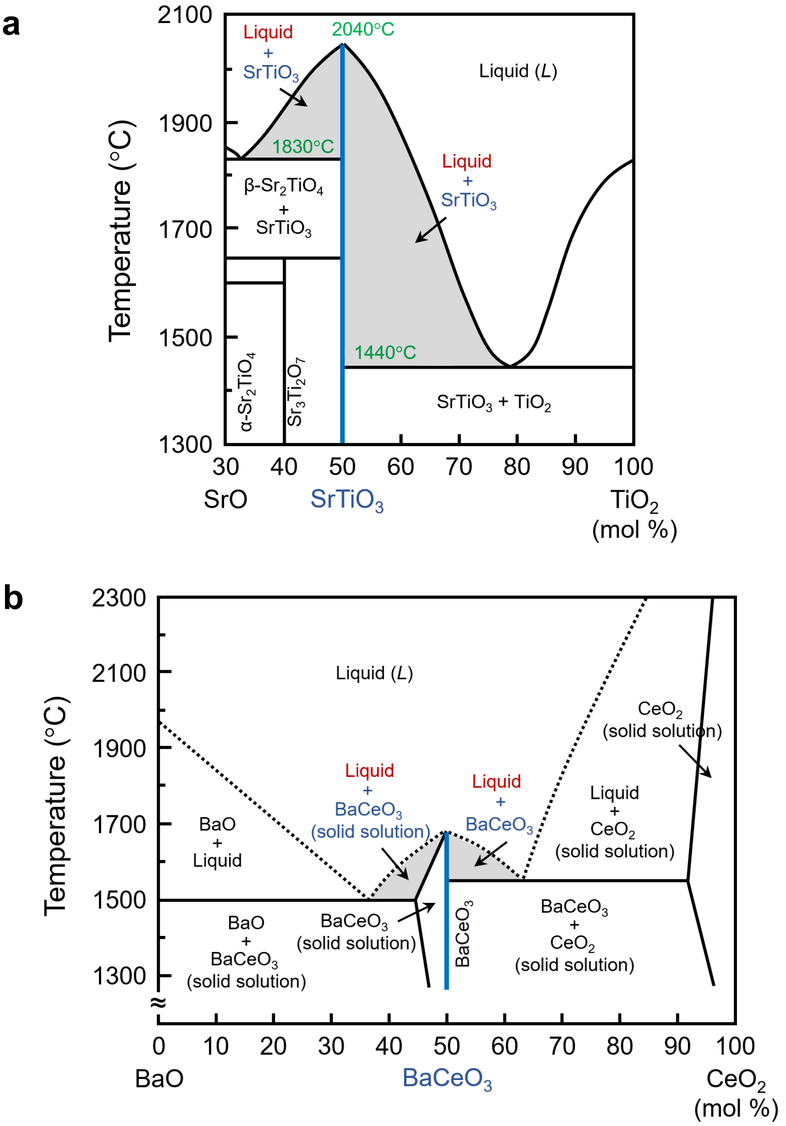

Supplementary Fig. 14 Phase diagrams of $SrTiO_3$ and $BaCeO_3$. (**a**) This diagram was reconstructed from the information in ref. 49. The $SrTiO_3$–liquid two-phase regions are denoted by a gray shadow. The formation of a liquid phase in these regions is induced by Sr/Ti nonstoichiometrty via either a Sr-rich or Ti-rich composition. (**b**) This diagram was reconstructed from the information in ref. 39, although different versions of the diagram for phase equilibrium can be found in previous reports. The $BaCeO_3$–liquid two-phase regions are also denoted by a gray shadow in this diagram for $BaCeO_3$.
