# Supplementary material for: Atomic-scale observation of premelting at 2D lattice defects inside oxide crystals
Source: Nat Commun. 2023 Apr 20;14:2255. doi: 10.1038/s41467-023-37977-w (PMC10119109; doi:10.1038/s41467-023-37977-w)
Supplement: Supplementary file 2 — Description of Additional Supplementary Files [file 41467_2023_37977_MOESM2_ESM.pdf]

Supplementary Movie 1

Video clip demonstrating the recrystallization of an amorphous layer by e-beam irradiation in STEM (× 2 speed).
